# Influence of Sewing Thread Properties on Seam Strength and Yielding Behavior in Woven Fabrics

**DOI:** 10.3390/ma19183864

**Published:** 2026-09-10

**Authors:** Anita Milosavljevic, Vasilije Petrovic, Jovana Stepanovic Profirovic, Dragan Djordjic, Marija Petrovic

**Affiliations:** 1 Technical Faculty “Mihajlo Pupin”, University of Novi Sad, 21000 Novi Sad, Serbia; anita.milosavljevic@uns.ac.rs (A.M.); vasilije.petrovic1962@gmail.com (V.P.); 2 Faculty of Technology, University of Niš, 18000 Niš, Serbia; stepanovicjovana@yahoo.com (J.S.P.); petrovic.marija.0808@gmail.com (M.P.); 3 Institute of General and Physical Chemistry, 11158 Belgrade, Serbia

**Keywords:** seam yielding point, seam strength, woven fabrics, sewing thread, force–elongation curve, seam mechanics

## Abstract

**Highlights:**

- The Seam Yielding Point (SYP) was identified as a reproducible indicator of the onset of seam deterioration.
- Seam yielding force and ultimate seam strength increased with sewing thread strength and fabric compactness.
- Force–elongation curves revealed progressive structural degradation prior to ultimate seam rupture.
- A strong positive relationship was observed between seam yielding force and seam breaking force.
- The proposed SYP provides complementary information for seam evaluation and may support optimization of thread selection and seam design.

**Abstract:**

The mechanical performance of sewn seams is commonly evaluated using parameters such as seam strength and seam efficiency, which primarily describe the behavior of the seam at ultimate failure. However, these parameters provide only limited information about the initiation of structural deterioration that precedes rupture. An operational definition of the Seam Yielding Point (SYP) is proposed as an experimentally identifiable indicator of the onset of seam degradation during tensile loading. Nine woven fabric variants differing in structural characteristics were sewn using four polyester sewing threads with different linear densities. Seam tensile tests were performed according to the relevant international standard, while force–elongation curves were continuously recorded. The SYP was identified as the first local decrease in force observed on the force–elongation curve. Photographic documentation was used only for qualitative interpretation of the deformation process. The experimental results showed that the proposed SYP could be consistently identified in all tested specimens prior to ultimate seam failure. The corresponding yielding force and elongation depended on both the sewing thread properties and the fabric structure. In addition to conventional seam strength measurements, the proposed approach provides valuable information regarding the initiation of irreversible structural changes within the seam. Overall, the proposed operational definition of the SYP represents a useful complementary parameter for evaluating seam mechanical behavior and contributes to a more comprehensive assessment of seam performance.

## 1. Introduction

The mechanical performance of sewn textile products is largely determined by the quality and integrity of their seams, which represent the primary structural elements responsible for joining individual fabric components into a functional assembly. During service, seams are continuously exposed to tensile, cyclic, and multidirectional loading, making their mechanical behavior one of the key factors governing garment durability, dimensional stability, functionality, and overall service life. Consequently, seam quality has become one of the most important criteria for evaluating the performance of apparel, technical textiles, protective clothing, and other sewn products. The mechanical response of a seam is influenced by numerous interacting factors, including fabric structure, sewing thread properties, stitch type, stitch density, sewing parameters, and the compatibility between the constituent materials. Achieving reliable seam performance therefore requires an appropriate combination of these parameters to ensure adequate load transfer, resistance to deformation, and structural integrity throughout the service life of the product [1,2,3,4]. Previous studies have also reported that sewing-induced damage and thread recovery behavior may additionally influence seam appearance and long-term mechanical performance [5,6].

The mechanical behavior of a seam is strongly influenced by the properties of the sewing thread, which serves as the primary load-bearing component within the stitched assembly. During the sewing process, the thread is subjected to repeated tensile loading, bending, compression, torsion, friction against machine elements, and interaction with the fabric structure. These mechanical actions may alter its original properties and consequently affect seam performance. Parameters such as thread linear density, fiber composition, tensile strength, elongation at break, surface characteristics, and structural stability have been shown to influence the load-carrying capacity, deformation behavior, and durability of seams under service conditions [7,8,9].

In addition to sewing thread characteristics, seam performance is affected by the compatibility between the thread and fabric, as well as by processing conditions including needle selection, thread tension, stitch density, sewing speed, and subsequent finishing treatments. Recent studies have further demonstrated that textile-processing operations, such as dyeing and finishing, may modify fabric mechanical properties and consequently influence seam strength and seam efficiency, emphasizing the complex interaction between material properties and manufacturing parameters [10,11,12,13].

The tensile behavior of a seam cannot be explained solely by the properties of the sewing thread or the fabric, but rather by the interaction of all components forming the sewn assembly. Under external loading, stresses are continuously redistributed between the sewing thread, the fabric yarns, and the stitch geometry, resulting in a complex mechanical response that evolves throughout the loading process [1,3,4,9,14]. Before ultimate failure occurs, localized structural changes such as yarn slippage, stitch deformation, thread straightening, and progressive stress redistribution may already develop within the seam [15,16]. The extent and sequence of these phenomena depend on the compatibility of the constituent materials, seam geometry, stitch configuration, and loading conditions. Consequently, seam performance should be interpreted as the combined mechanical response of the entire sewn structure rather than as the consequence of a single material property or manufacturing parameter. A comprehensive understanding of these interactions is therefore essential for accurately characterizing seam behavior and improving the reliability and durability of sewn products [4,9,10,17,18].

Previous research has substantially improved the understanding of seam behavior by investigating various aspects of seam performance under mechanical loading. Considerable attention has been devoted to evaluating seam strength and seam efficiency as the principal indicators of seam quality, while numerous studies have examined seam slippage, seam puckering, and stitch failure mechanisms under different loading conditions [14,15,16,17,18,19]. Other investigations have focused on the influence of sewing parameters, including stitch density, seam type, needle characteristics, sewing thread properties, and fabric structure, demonstrating that even relatively small variations in these parameters may significantly affect the mechanical response of sewn assemblies [15]. More recent studies have additionally reported that textile finishing and processing treatments may alter the mechanical properties of fabrics and consequently influence seam performance by modifying the interaction between the sewing thread and the fabric during tensile loading [16]. Collectively, these investigations have established a solid understanding of the factors governing seam strength, seam slippage, seam deformation, and overall seam durability, providing valuable guidelines for optimizing sewing processes and improving the mechanical performance of sewn textile products [20,21,22,23,24].

Although the aforementioned studies have significantly advanced the understanding of seam mechanical behavior, they predominantly characterize seam performance by parameters measured at or near ultimate failure, such as seam strength, seam efficiency, or seam slippage resistance. In practical applications, however, seam deterioration rarely occurs as an instantaneous event. Instead, irreversible structural changes generally develop progressively through localized yarn displacement, stitch deformation, gradual stress redistribution, and partial loss of structural integrity while the seam is still capable of carrying increasing tensile loads. Consequently, the transition between elastic seam behavior and the onset of irreversible structural deterioration remains insufficiently characterized by conventional seam evaluation methods. The experimental identification of this transition may therefore provide valuable complementary information regarding seam mechanical behavior and improve the understanding of damage evolution before complete seam failure occurs.

Accordingly, the objective of this study was not only to evaluate the influence of sewing thread properties on seam mechanical behavior but also to introduce and experimentally validate the Seam Yielding Point as a complementary operational parameter derived from force–elongation curves.

To the best of the authors’ knowledge, previous studies have primarily evaluated seam performance using breaking force, seam efficiency, seam slippage, or seam puckering, whereas no operational parameter describing the onset of localized seam yielding directly from force–elongation curves has been explicitly reported.

## 2. Materials and Methods

### 2.1. Materials

Nine woven fabrics commonly used for apparel manufacturing were selected for this study. The fabrics differed in their structural characteristics to investigate the influence of sewing thread properties on seam behavior under tensile loading. Four commercially available 100% polyester sewing threads (Coats Astra) with different linear densities and mechanical properties were used for seam preparation. Their structural and mechanical characteristics, including fineness, breaking force, breaking elongation, and recommended needle fineness, are presented in Table 1.The principal characteristics of the investigated fabrics, including fiber composition, weave structure, yarn linear density, thread density, mass per unit area, and mechanical properties, are summarized in Table 2. All investigated fabrics were woven from 100% cotton yarns in a plain weave (1/1) structure. The warp yarn linear density was maintained at 40 tex, while the warp density was kept constant at 24 ends cm^−1^ for all specimens. To obtain fabrics with different structural characteristics, three weft yarn linear densities (20, 30, and 50 tex) and three weft densities (10, 17, and 25 picks cm^−1^) were employed, resulting in nine woven fabric variants (A–I). Warp and weft densities were determined according to EN 1049-2 [25], yarn linear density according to SRPS ISO 7211-5 [26], and fabric mass per unit area according to SRPS EN 12127 [27].

**Table 1:** Mechanical and structural characteristics of the sewing threads used in the study.

Thread Type	Raw Material Composition (%)	Yarn Count (Nm)	Breaking Force (cN)	Breaking Elongation (%)	Fineness of the Needle Used (Nm)
K1	100 PES	120	1099	19	80
K2		100	1280	17	80
K3		75	1800	15	100
K4		50	2400	14	110

**Table 2:** Structural characteristics of the woven fabric variants investigated in this study.

Sample Marking	Weave Structure	Linear Density T_t_ (tex)		Yarn Type (%)		Thread Density		Mass Per Unit Area (g⋅m^−2^)
		for Warp	for Weft	for Warp	for Weft	Warp d_wa_ (cm^−1^)	Weft d_we_ (cm^−1^)	
A	Plain 1/1	40	50	100 Co	100 Co	24	25	232.52
B	Plain 1/1	40	30	100 Co	100 Co	24	25	190.32
C	Plain 1/1	40	20	100 Co	100 Co	24	25	153.40
D	Plain 1/1	40	50	100 Co	100 Co	24	17	175.77
E	Plain 1/1	40	30	100 Co	100 Co	24	17	154.45
F	Plain 1/1	40	20	100 Co	100 Co	24	17	132.30
G	Plain 1/1	40	50	100 Co	100 Co	24	10	153.40
H	Plain 1/1	40	30	100 Co	100 Co	24	10	136.52
I	Plain 1/1	40	20	100 Co	100 Co	24	10	121.75

### 2.2. Preparation of Specimens

Seams were sewn in the warp direction using seam type 1.01.01 according to ISO 4916:1991 [28] and lockstitch type 301 according to ISO 4915 [29]. Sewing was performed on a universal sewing machine (Juki DDL-5550, Juki Corporation, Tokyo, Japan) using lockstitch type 301. The following machine settings were applied: Stitching speed: 3000 stitches/min; stitch density—6 stitches/cm; presser foot pressure: 2.8 N; and thread tension: 0.50 N. The test samples were sewn using threads produced by Coats Astra (London, UK) [30], whose properties are presented in Table 1.

The selected fabrics were intended to provide a broad range of structural characteristics representative of woven apparel materials used in industrial garment manufacturing.

Test specimens were prepared in accordance with ISO 13935-1 [31] for determining the seam strength of sewn fabrics. Prior to sewing and mechanical testing, all fabrics and sewing threads were conditioned under standard atmospheric conditions for textile testing. Rectangular specimens measuring 350 × 700 mm were cut from each woven fabric variant with the longer dimension oriented in the weft direction. Each specimen was folded lengthwise so that the fold line remained parallel to the longer side of the specimen prior to sewing. Seams were produced parallel to the warp direction using lockstitch type 301 (1.01.01/301) in accordance with ISO 4916. Sewing was performed on a Juki DDL-5550 industrial lockstitch sewing machine (Juki Corporation, Tokyo, Japan). To eliminate the influence of sewing conditions on seam behavior, identical machine settings were maintained throughout the investigation, including a sewing speed of 3000 stitches min^−1^, a stitch density of 6 stitches cm^−1^, a presser foot pressure of 2.8 N, and a thread tension of 0.50 N. Only the sewing thread type was varied according to the experimental design.

This experimental design enabled the systematic and isolated evaluation of the influence of sewing thread properties on seam mechanical behavior.

**Figure 1:**
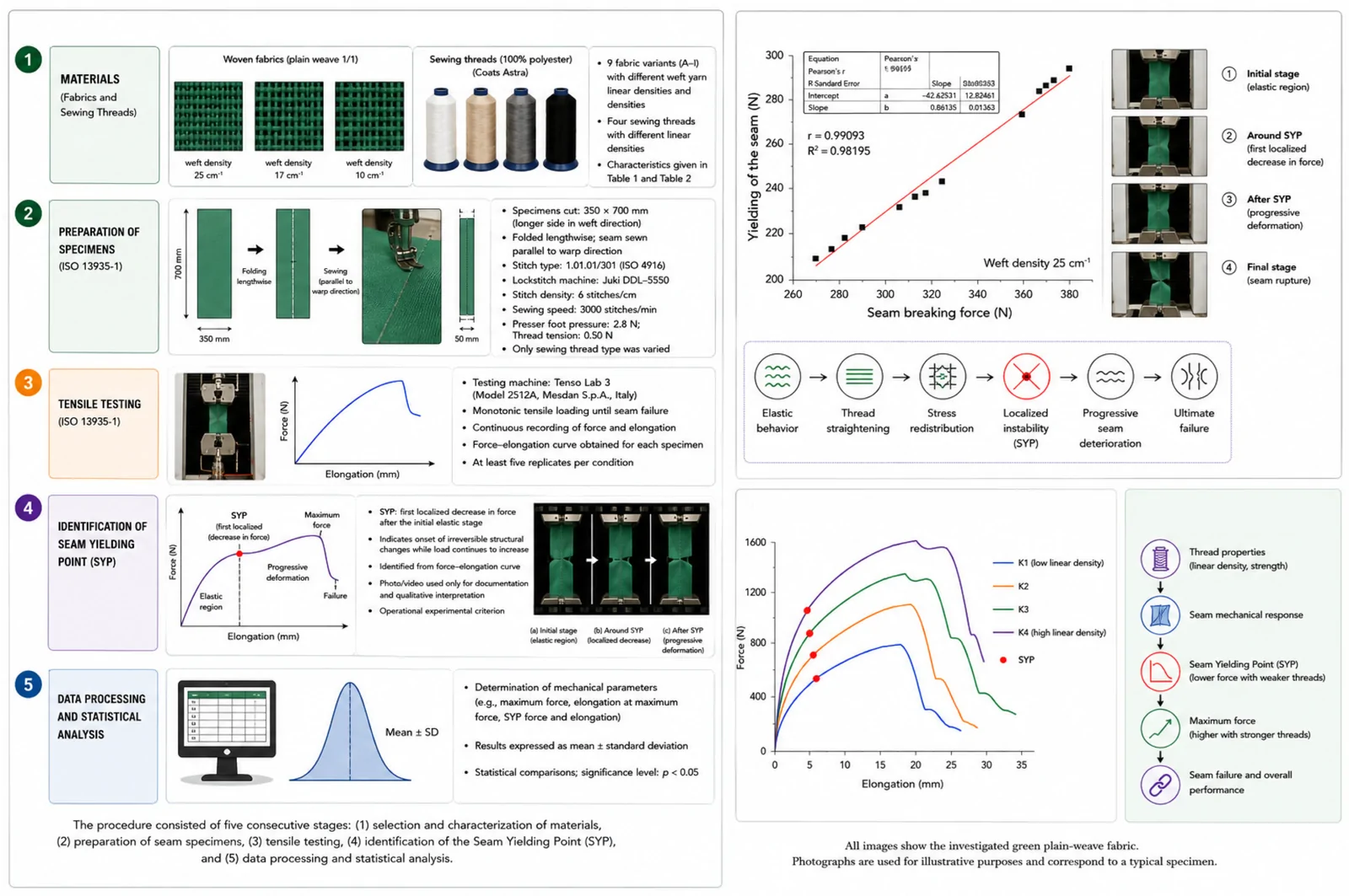
Experimental workflow adopted in this investigation. The methodology consisted of five consecutive stages: (1) selection and characterization of woven fabrics and sewing threads, (2) preparation of seam specimens according to ISO 13935-1, (3) tensile testing and acquisition of force–elongation curves, (4) identification of the Seam Yielding Point (SYP) as the first localized decrease in tensile force associated with the onset of irreversible structural changes, and (5) determination of characteristic mechanical parameters and subsequent statistical analysis.

The overall experimental workflow adopted in this study is schematically illustrated in Figure 1, summarizing the sequence of specimen preparation, tensile testing, identification of the Seam Yielding Point (SYP), and statistical data processing.

Photographic documentation was used exclusively for qualitative interpretation of the deformation process.

### 2.3. Tensile Testing

**Figure 2:**
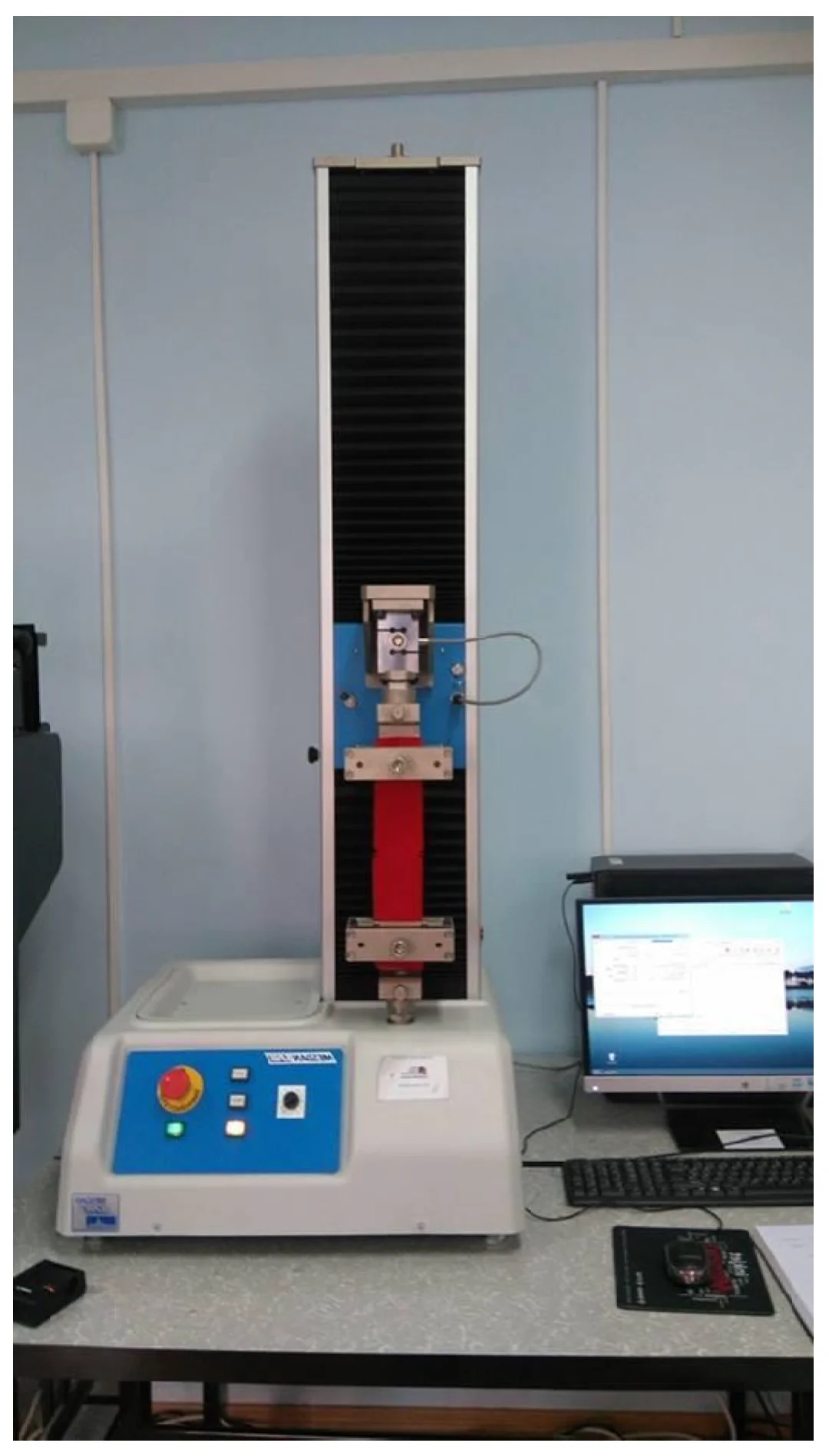
Experimental setup used for seam tensile testing on the Tenso Lab 3 universal tensile testing machine (Mesdan S.p.A., Italy) according to ISO 13935-1.

Mechanical testing was performed using a Tenso Lab 3 universal tensile testing machine (Model 2512A, Mesdan S.p.A., Raffa, Italy) in accordance with the requirements of ISO 13935-1. Each specimen was mounted between the machine grips and loaded under monotonic tensile loading until complete seam failure. The loading rate and gauge length were selected in accordance with ISO 13935-1. All tests were performed under identical environmental conditions after specimen conditioning. The experimental setup used for seam tensile testing is presented in Figure 2.

The recorded force–elongation curves served as the basis for determining all characteristic mechanical parameters analyzed in this study.

For each experimental condition, at least five replicate specimens were tested, and the reported values represent arithmetic means.

The recorded force–elongation curves were subsequently used to identify the characteristic stages of seam deformation and the corresponding Seam Yielding Point (SYP). 

### 2.4. Identification of the Seam Yielding Point (SYP)

In addition to the conventional mechanical parameters obtained from the tensile tests, the present study introduces the Seam Yielding Point (SYP) as an operational experimental parameter for characterizing the onset of irreversible structural changes within the sewn assembly during tensile loading. For the purposes of this investigation, the Seam Yielding Point was operationally defined as the first localized decrease in tensile force observed on the force–elongation curve following the initial elastic loading stage. Unlike the maximum tensile force, which represents the ultimate load-carrying capacity of the seam, the SYP indicates the transition from predominantly elastic behavior to the initiation of irreversible structural changes while the seam continues to sustain increasing deformation. Minor force fluctuations occurring before the onset of a distinct force reduction were not considered. The identification of the SYP was based on detailed analysis of the recorded force–elongation curves. Photographic and video records acquired during testing were used exclusively for documentation of the deformation process and for qualitative interpretation of the observed failure mechanisms. Qualitative examination of the documented deformation process indicated that the occurrence of the SYP coincided with the initiation of localized structural changes, including thread straightening, stitch deformation, local yarn displacement, stress redistribution within the stitched assembly, and the onset of progressive structural instability preceding ultimate seam failure.

The proposed definition is intended as an operational experimental criterion developed for the present investigation rather than as a universal constitutive definition of material yielding. Accordingly, the SYP should be regarded as a reproducible experimental indicator for identifying the transition between elastic seam behavior and the onset of irreversible structural deterioration under monotonic tensile loading.

The developed experimental procedure allows the SYP to be identified consistently using experimentally recorded force–elongation curves without introducing additional mathematical modeling. The same operational definition was consistently applied to all investigated specimens.

**Figure 3:**
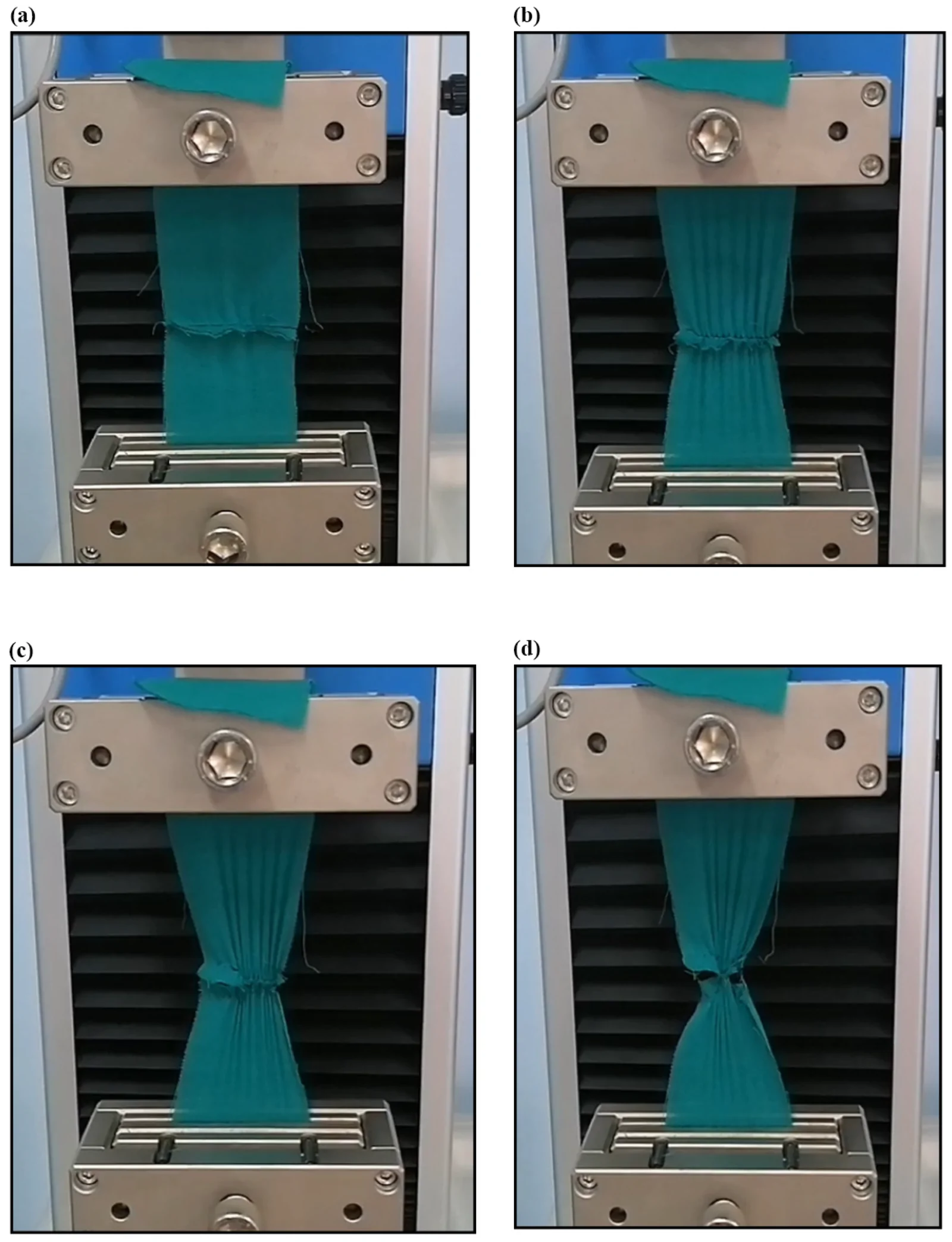
Representative sequence of seam deformation during tensile testing: (**a**) initial specimen before loading; (**b**) initiation of localized seam yielding corresponding to the experimentally identified Seam Yielding Point (SYP); (**c**) progressive propagation of seam yielding after the SYP; and (**d**) final seam rupture corresponding to the Breaking Point (BP). The photographs are presented exclusively for qualitative visualization of the deformation process, whereas the Seam Yielding Point was identified solely from the force–elongation curves.

Representative photographs illustrating the successive stages of seam deformation are presented in Figure 3. Although the occurrence of localized seam deformation observed in the photographs qualitatively coincided with the experimentally identified Seam Yielding Point, the SYP was determined exclusively from the recorded force–elongation curves. The photographic documentation served only as visual confirmation of the progressive deformation mechanism.

### 2.5. Data Processing and Statistical Analysis

All experimental results were processed using the recorded force–elongation data obtained from the tensile tests. The characteristic mechanical parameters, including maximum tensile force, elongation at maximum force, yielding force, and yielding elongation, were determined from the corresponding force–elongation curves. For each experimental group, the arithmetic mean and standard deviation were calculated. The mean values are presented in Table 3, Table 4 and Table 5. Linear regression and correlation analyses were additionally performed to evaluate the relationship between yielding force and maximum seam strength.

All force–elongation curves were visually inspected to ensure consistent identification of the Seam Yielding Point according to the operational criterion defined in Section 2.4.

## 3. Results

The mechanical behavior of the sewn specimens was evaluated by tensile testing in order to investigate the influence of sewing thread properties on seam yielding and ultimate seam strength. For all investigated fabric variants, the force–elongation curves exhibited a similar overall shape characterized by an initial elastic region, followed by the occurrence of the Seam Yielding Point (SYP), continued load-bearing after yielding, and finally complete seam rupture. The experimental results showed good consistency among the tested specimens, indicating satisfactory repeatability of the experimental procedure. The average values of seam breaking force, breaking elongation, yielding force, and yielding elongation obtained for the investigated fabric groups are summarized in Table 3, Table 4 and Table 5, corresponding to weft thread densities of 25, 17, and 10 cm^−1^.

### 3.1. Effect of Sewing Thread Properties on Seam Yielding and Breaking Behavior

Analysis of the experimental data presented in Table 3 revealed a consistent influence of sewing thread properties on the mechanical response of the investigated seams. Regardless of the fabric structure, increasing sewing thread strength was accompanied by higher seam yielding forces and higher ultimate seam breaking forces. This tendency indicates that stronger sewing threads delayed the onset of localized structural instability within the seam while simultaneously increasing its load-bearing capacity. As a result, both the yielding stage and the final rupture occurred at higher applied loads as the mechanical performance of the sewing thread increased.

For all investigated thread types, the relationship between yielding force and seam breaking force remained consistent. Although the absolute values varied among the individual fabric variants, the same tendency was observed throughout the experimental series. Fabrics produced with higher weft densities generally exhibited higher seam resistance than fabrics with lower weft densities, reflecting the beneficial contribution of increased fabric compactness to load transfer across the stitched assembly.

The experimental observations also showed that the influence of sewing thread properties extended beyond the ultimate seam strength. Comparable trends were also observed for the yielding force, suggesting that the proposed Seam Yielding Point (SYP) is sensitive to changes in sewing thread mechanical characteristics and may therefore serve as a complementary experimental parameter for evaluating seam performance prior to ultimate failure.

The numerical comparison supports these observations. For fabric group A, the seam breaking force increased from 357 N for thread K1 to 372 N for thread K4, while the corresponding yielding force increased from 282 N to 294 N. Similar increases were observed for fabric groups B and C, indicating that improvements in sewing thread mechanical performance affected both the onset of localized structural deterioration and the ultimate load-bearing capacity of the seam. Although the magnitude of these increases differed among the investigated fabric variants, the direction of the effect remained unchanged throughout the experimental series.

In addition to sewing thread properties, fabric structure also played an important role in determining seam behavior. At identical sewing thread types, fabrics with higher weft densities generally exhibited higher yielding and breaking forces than fabrics with lower weft densities, indicating more efficient stress transfer within the sewn assembly. This effect became particularly evident when comparing corresponding fabric groups presented in Table 3, Table 4 and Table 5, confirming that seam performance is governed by the combined mechanical response of both the sewing thread and the woven fabric.

For fabrics with a weft density of 17 cm^−1^, the obtained results confirmed (Table 4) the trends observed for the denser fabric group. The characteristic stages of seam deformation observed during tensile testing are illustrated in Figure 3. The photographs clearly demonstrate that localized structural deterioration develops progressively and does not immediately result in complete seam rupture. Increasing sewing thread strength consistently resulted in higher yielding and breaking forces for all investigated fabric variants. However, compared with fabrics having a weft density of 25 cm^−1^, slightly lower force values were generally recorded, indicating that the reduction in fabric compactness decreased the ability of the stitched assembly to redistribute tensile stresses efficiently. Nevertheless, the proportional increase associated with stronger sewing threads remained comparable, demonstrating that the influence of sewing thread properties was largely independent of fabric density.

A further reduction in weft density to 10 cm^−1^ produced the lowest yielding and breaking forces among all investigated fabric groups (Table 5). The larger spacing between adjacent weft yarns reduced the structural compactness of the woven fabric, thereby limiting the capability of the seam to transfer tensile loads effectively. Despite this reduction in absolute force values, the beneficial influence of increasing sewing thread strength remained evident throughout the entire experimental series, confirming that both seam yielding and ultimate seam strength were simultaneously governed by sewing thread properties and fabric structural characteristics.

### 3.2. Force–Elongation Behavior and Identification of the Seam Yielding Point

**Figure 4:**
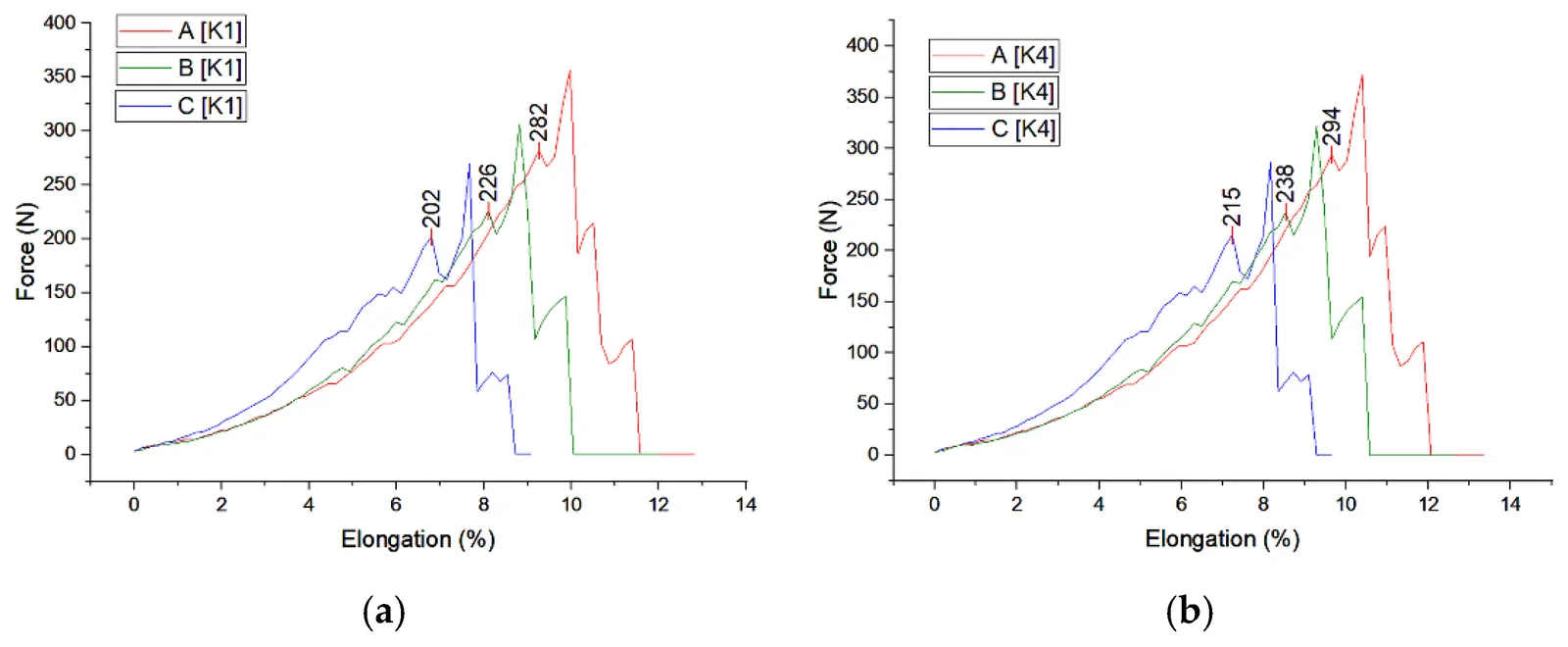
Representative force–elongation curves for specimens with a weft density of 25 cm^−1^ shown for thread types: (**a**) K1 and (**b**) K4.

**Figure 5:**
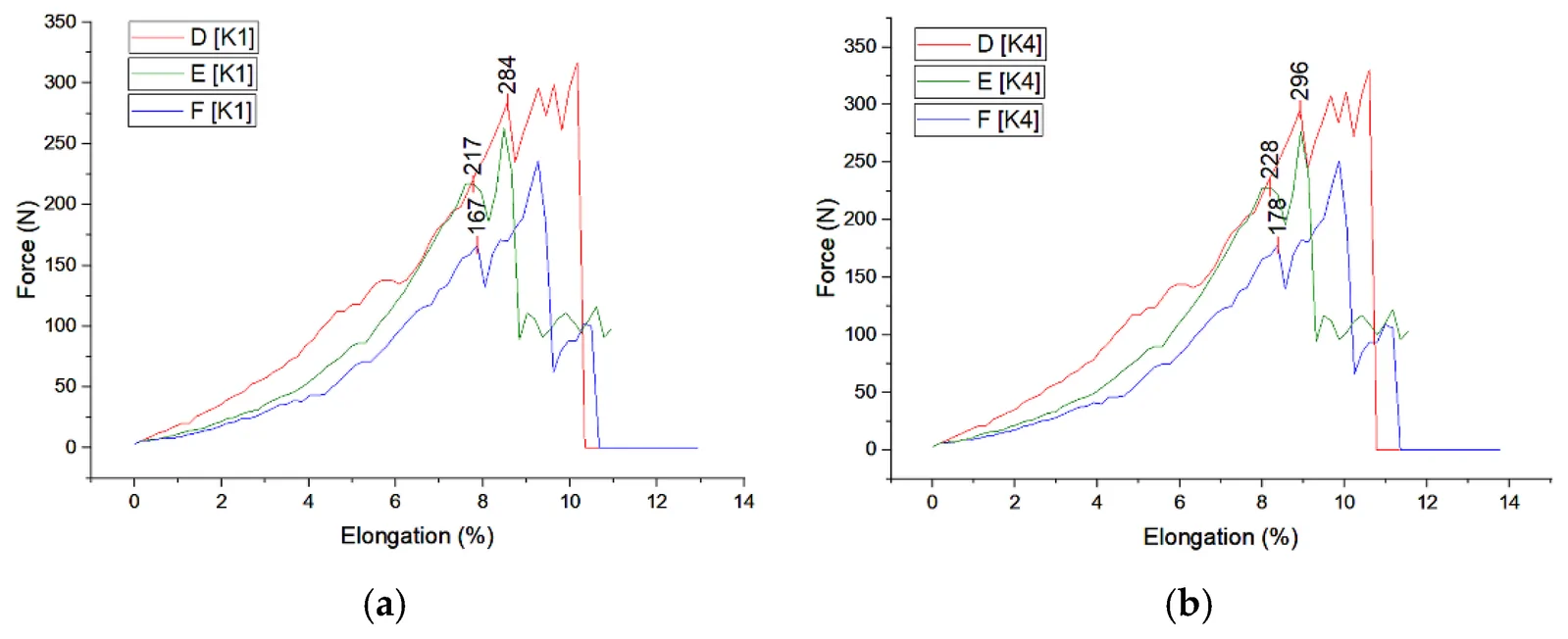
Representative force–elongation curves for specimens with a weft density of 17 cm^−1^, shown for thread types: (**a**) K1 and (**b**) K4.

**Figure 6:**
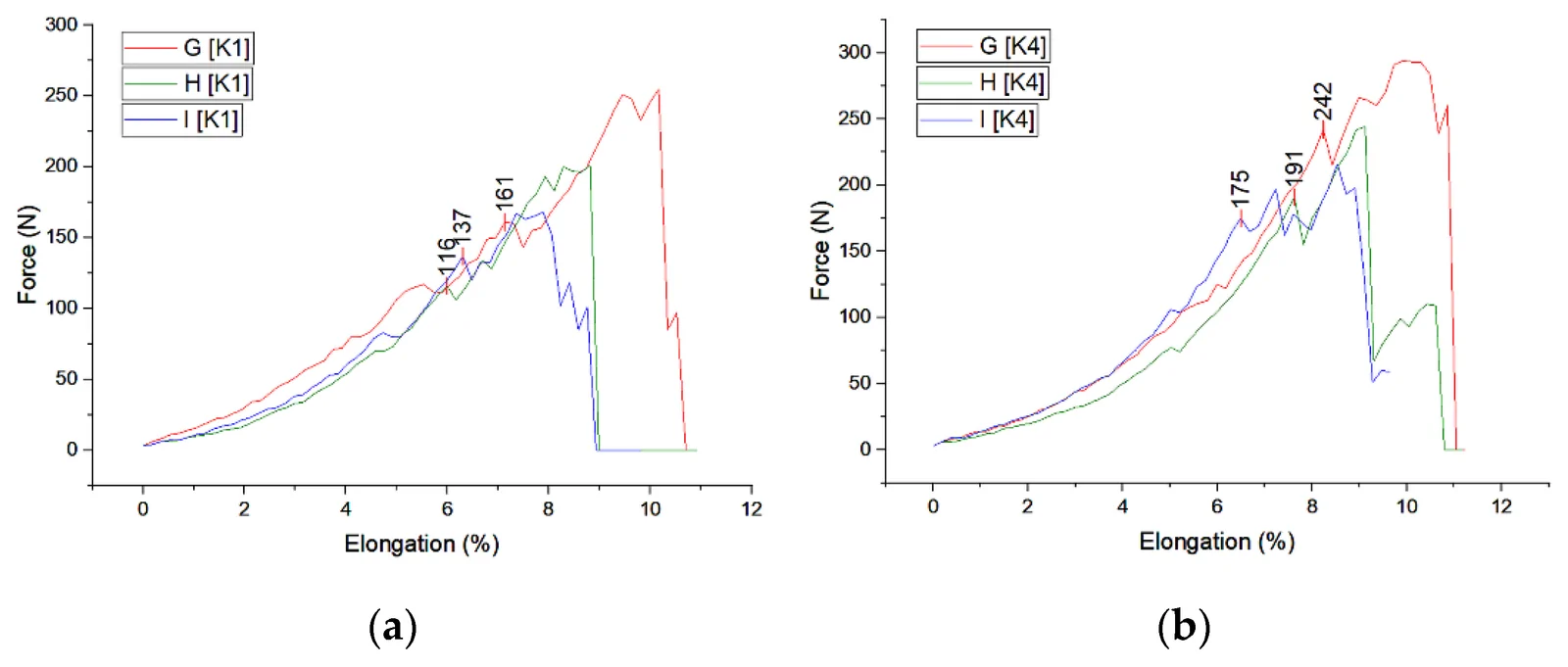
Representative force–elongation curves for specimens with a weft density of 10 cm^−1^, shown for thread types: (**a**) K1 and (**b**) K4.

Representative force–elongation curves for the investigated seam assemblies are shown in Figure 4, Figure 5 and Figure 6. Despite differences in fabric structure and sewing thread properties, all specimens exhibited a similar sequence of deformation stages. Initially, the applied tensile force increased almost linearly, corresponding to the predominantly elastic response of the sewn assembly. As loading continued, the curves reached a characteristic point marked by a localized decrease in force, identified in this study as the Seam Yielding Point (SYP). Following this temporary force reduction, the specimens remained capable of sustaining additional tensile loading until the maximum seam breaking force was reached.

The appearance of the SYP shows that irreversible structural changes begin well before complete seam rupture occurs. Instead, the temporary reduction in force reflects local stress redistribution within the stitched assembly, after which the remaining structural components continue to transfer the applied load. Accordingly, the force–elongation curves illustrate that seam degradation develops progressively rather than instantaneously, allowing the seam to retain a substantial portion of its load-bearing capacity even after the onset of localized structural instability.

Although the general shape of the force–elongation curves remained similar for all investigated specimens, noticeable differences in both the magnitude of the yielding force and the subsequent load-bearing behavior were observed. Specimens sewn with stronger sewing threads generally exhibited higher yielding forces, higher maximum seam strengths, and smoother post-yield deformation, whereas weaker sewing threads produced earlier yielding and lower ultimate load capacities. These findings are in agreement with the experimental data summarized in Table 3, Table 4 and Table 5 and confirm that sewing thread mechanical properties influence not only seam strength but also the entire deformation process leading to failure.

The consistent appearance of the SYP in all investigated specimens demonstrates that the proposed experimental criterion can be consistently identified regardless of the investigated fabric variant. Although the absolute force values depended on fabric structure and sewing thread properties, the characteristic sequence consisting of elastic loading, localized yielding, continued load-bearing, and ultimate rupture remained common to all tested seam assemblies.

The representative force–elongation curves further demonstrate that the Seam Yielding Point (SYP) does not correspond to catastrophic seam failure but rather to the onset of localized structural deterioration. After the occurrence of the SYP, all investigated specimens retained a measurable load-bearing capacity, as evidenced by the subsequent increase in tensile force before ultimate rupture. This behavior indicates that the stitched assembly continues to redistribute stresses among the sewing thread, fabric yarns, and stitch geometry despite the initiation of irreversible structural changes. The interval between the SYP and the breaking point represents a progressive damage development stage rather than an abrupt transition to complete failure.

### 3.3. Relationship Between Seam Yielding Force and Seam Breaking Force

**Figure 7:**
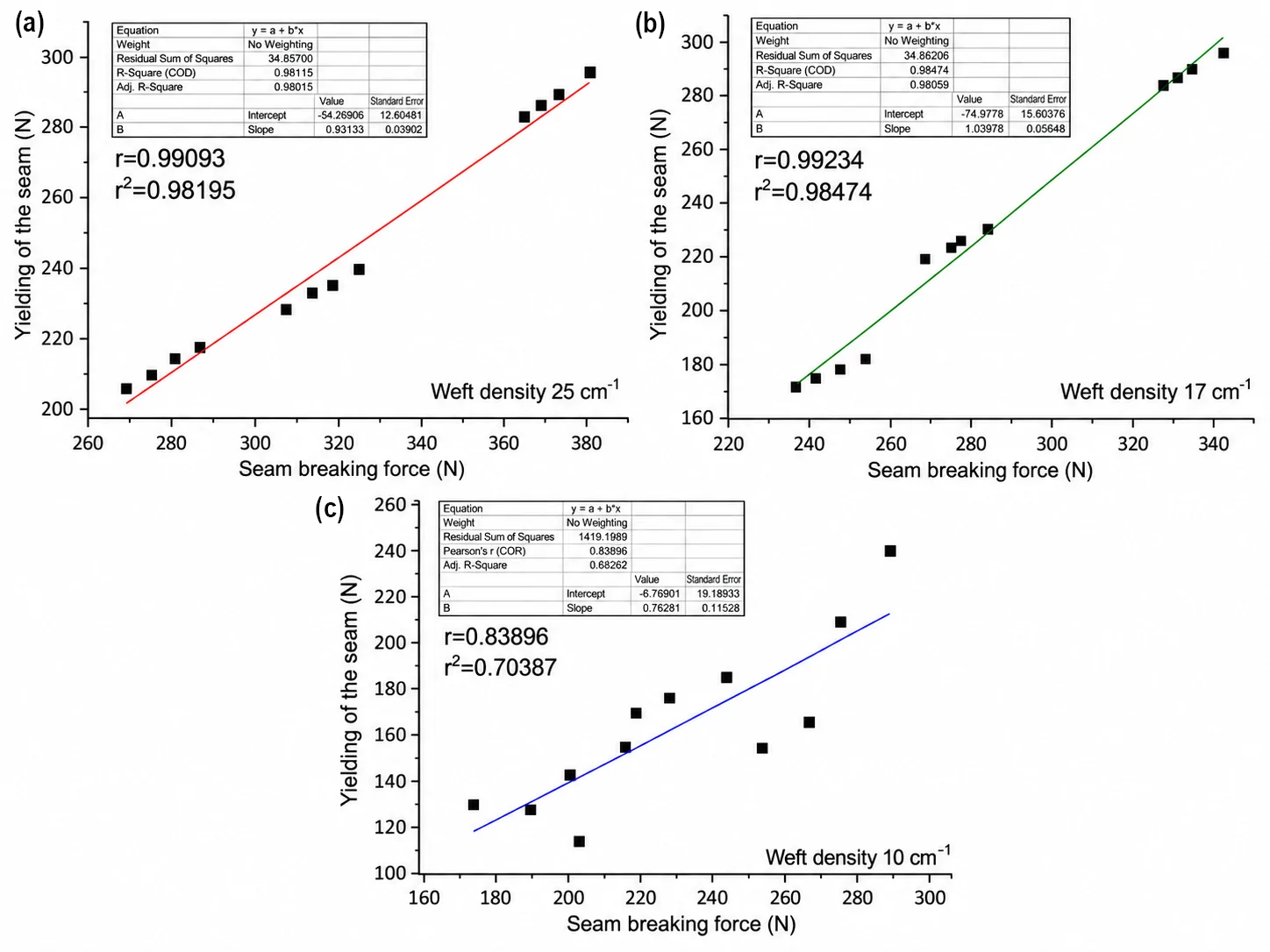
Linear relationship between the experimentally determined Seam Yielding Force and the corresponding Seam Breaking Force for woven fabrics with weft densities of (**a**) 25 cm^−1^, (**b**) 17 cm^−1^, and (**c**) 10 cm^−1^. Black squares represent experimental data, while the solid lines indicate the corresponding linear regression fits.

Linear regression was used to evaluate the relationship between the seam yielding force and the corresponding seam breaking force for woven fabrics differing in weft density (Figure 7). In all investigated fabric groups, a strong positive linear relationship was observed, indicating that specimens exhibiting higher yielding forces also developed higher ultimate seam strengths. The corresponding coefficients of determination (R^2^) exceeded 0.70 for all investigated fabric structures and approached 1.0 for fabrics with higher structural compactness, confirming the strong relationship between the onset of localized structural deterioration and the final load-bearing capacity of the seam. The strongest correlation was observed for fabrics with a weft density of 25 cm^−1^, where the regression model demonstrated an almost perfect linear relationship between yielding force and seam breaking force. A similarly high correlation was obtained for fabrics with a weft density of 17 cm^−1^, whereas a moderate reduction in the coefficient of determination was observed for the least compact fabric structure (10 cm^−1^). The lower correlation obtained for the latter group can be attributed to the increased structural variability associated with the larger spacing between adjacent weft yarns, which resulted in greater variability of stress redistribution during tensile loading.

Despite the observed differences, all regression models followed the same overall trend, namely that the seam yielding force increased proportionally with the seam breaking force. This consistency confirms that the proposed Seam Yielding Point (SYP) represents a stable experimental characteristic of seam mechanical behavior and supports its use as a complementary parameter for describing the progressive development of seam deterioration.

### 3.4. Quantitative Assessment of the Seam Yielding Point

**Figure 8:**
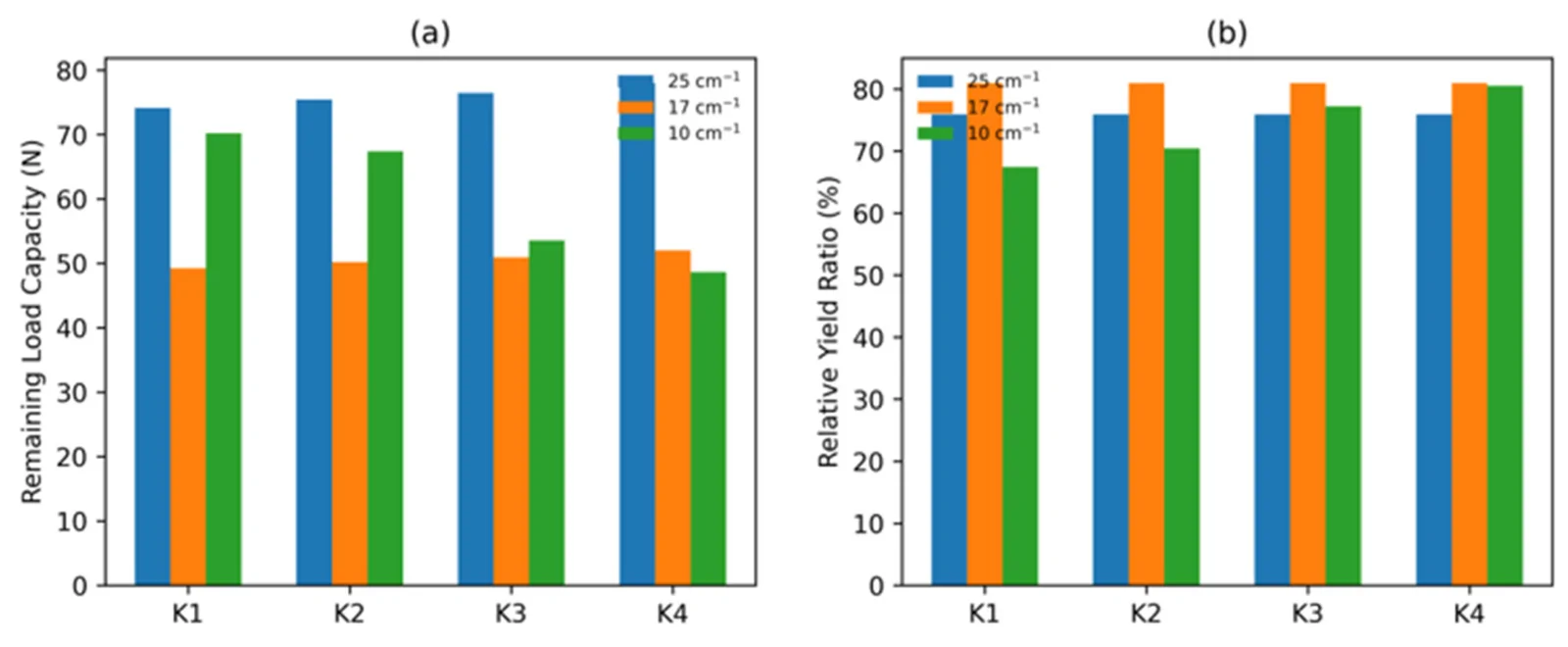
Quantitative evaluation of the proposed Seam Yielding Point using the derived Remaining Load Capacity (RLC) and Relative Yield Ratio (RYR) parameters for stitched woven fabrics with different weft densities. Panel (**a**) presents the average Remaining Load Capacity, while panel (**b**) shows the average Relative Yield Ratio for sewing threads K1–K4.

To further evaluate the engineering significance of the proposed Seam Yielding Point (SYP), two additional quantitative parameters were derived from the experimental results: the Remaining Load Capacity (RLC), defined as the difference between the seam breaking force and the yielding force (RLC = FBP − FSYP), and the Relative Yield Ratio (RYR), defined as the ratio between the yielding force and the ultimate breaking force (RYR = FSYP/FBP × 100). The Remaining Load Capacity (RLC) quantifies the additional tensile load sustained by the seam after the onset of localized structural deterioration. Unlike the conventional breaking force, which represents the final failure event, RLC characterizes the progressive damage stage that exists between the first detectable structural degradation and complete rupture. The calculated RYR values varied with fabric structure. The highest mean RYR was observed for fabrics with a weft density of 17 cm^−1^, while the lowest mean RYR was obtained for the least compact fabric group (10 cm^−1^). These results indicate that the relative position of the SYP with respect to the ultimate breaking force depends on fabric structure and does not vary monotonically with weft density. Similarly, the calculated RLC values revealed that seams retained a substantial portion of their load-bearing capacity after the occurrence of the SYP. The calculated values presented in Figure 8 and Table 6 highlight these relationships, demonstrating that the proposed SYP provides additional engineering information beyond conventional seam breaking force measurements.

Remaining Load Capacity (RLC) = Breaking force − Seam Yielding Force Relative Yield Ratio (RYR) = (Seam Yielding Force/Breaking Force) × 100. Values are arithmetic means calculated from all investigated sewing thread types (K1–K4).

The data summarized in Table 6 show that fabrics with higher structural compactness generally exhibited higher seam yielding forces and higher breaking forces. In contrast, the RLC and RYR values varied with fabric structure, indicating that the post-yield mechanical response cannot be explained solely by weft density. Nevertheless, all investigated seam groups retained a measurable load-bearing capacity after the occurrence of the Seam Yielding Point.

Overall, stronger sewing threads increased the seam yielding force, whereas the remaining load capacity varied depending on both sewing thread type and fabric structure. The values shown in Figure 8 represent arithmetic means calculated for each sewing thread type across the corresponding fabric variants within each weft-density group.

As shown in Table 6 and Figure 8, the investigated seams retained a measurable load-bearing capacity after the occurrence of the Seam Yielding Point. Across the individual fabric–thread combinations, the Remaining Load Capacity ranged from 31 to 99 N, depending on fabric structure and sewing thread type. The Relative Yield Ratio varied with fabric structure rather than following a strictly monotonic trend with weft density. The highest mean RYR was observed for fabrics with a weft density of 17 cm^−1^, while the lowest mean RYR was obtained for the least compact fabric group (10 cm^−1^). 

Overall, the derived RLC and RYR parameters demonstrate that stitched seams retain a measurable load-bearing capacity after the occurrence of the SYP, providing complementary engineering information that cannot be obtained from conventional seam strength measurements alone.

## 4. Discussion

The present findings indicate that seam mechanical behavior cannot be fully characterized solely by the ultimate seam strength. Although the maximum breaking force remains the conventional parameter for evaluating seam performance, the force–elongation curves obtained in the present investigation reveal that irreversible structural changes begin considerably earlier during tensile loading. The identified Seam Yielding Point (SYP) consistently preceded ultimate rupture in all investigated specimens, indicating that seam deterioration develops progressively through localized structural rearrangements rather than by instantaneous failure. These findings provide additional insight into the evolution of seam damage and extend the information obtained from conventional seam strength measurements.

The obtained results consistently indicate that sewing thread properties influence both seam yielding and ultimate seam strength. Increasing sewing thread strength resulted in higher yielding forces and higher breaking forces for all investigated fabric structures. This finding can be explained by the fact that stronger sewing threads delay the initiation of localized structural deterioration while simultaneously increasing the load-bearing capacity of the stitched assembly [5,6]. Consequently, the mechanical characteristics of the sewing thread affect not only the final failure resistance of the seam but also the entire deformation process preceding rupture.

Fabric architecture also had a pronounced influence on seam performance. Fabrics with higher weft densities generally developed higher yielding and breaking forces, reflecting their greater structural compactness and improved ability to redistribute tensile stresses within the stitched assembly. Increasing fabric compactness enhances frictional interaction between adjacent yarns, resulting in more efficient stress transfer and delayed development of localized deformation. Conversely, fabrics with lower weft densities exhibited lower resistance because larger inter-yarn spacing reduced the structural stability of the woven system during loading.

Following the occurrence of the Seam Yielding Point, the investigated seam assemblies continued to sustain increasing tensile loads before ultimate rupture. This behavior demonstrates that localized structural deterioration does not immediately compromise the load-bearing capacity of the seam. Instead, stronger sewing threads retained sufficient residual mechanical integrity to facilitate further stress redistribution within the stitched assembly, thereby delaying complete structural failure. Such behavior agrees well with the progressive nature of seam failure, in which irreversible structural changes initially remain localized before propagating through the stitched assembly. Consequently, the interval between the SYP and ultimate rupture represents a mechanically meaningful stage that is not described by conventional seam strength measurements.

Although minor differences were observed among individual fabric variants, the overall experimental trends remained highly consistent throughout the investigation, confirming that the relationships between sewing thread properties, fabric structure, and seam mechanical behavior were robust.

From a mechanical standpoint, the obtained behavior can be attributed to interactions between sewing thread properties and fabric architecture during tensile loading. Stronger sewing threads delay localized deformation and improve stress redistribution, while increased fabric compactness enhances frictional interaction between adjacent yarns, resulting in more efficient load transfer. These observations are consistent with the quantitative trends summarized by the Remaining Load Capacity and Relative Yield Ratio. Consequently, seam performance should be interpreted as the mechanical response of the entire sewn assembly rather than as the consequence of an individual material property.

Although the investigated fabrics differed in weft density and yarn linear density, the same sequence of mechanical events was consistently observed for all seam assemblies. The occurrence of the Seam Yielding Point, followed by continued load-bearing and ultimate rupture, indicates that the proposed operational definition is robust with respect to the investigated structural variations. This consistency supports the applicability of the SYP as a reproducible experimental characteristic of seam mechanical behavior rather than a phenomenon restricted to a particular fabric configuration.

The regression analysis demonstrated a strong positive relationship between the seam yielding force and the corresponding seam breaking force for all investigated fabric structures. Although the coefficient of determination decreased slightly with decreasing fabric compactness, the observed correlations remained consistently high, confirming that the proposed Seam Yielding Point represents a stable experimental characteristic of seam mechanical behavior. These findings support the applicability of the SYP as a complementary parameter for evaluating the progressive development of seam deterioration prior to ultimate failure. The consistently high correlations further indicate that the proposed SYP can be reproducibly identified across different fabric structures without relying solely on ultimate failure measurements.

The introduction of the Seam Yielding Point extends the conventional characterization of seam performance by providing additional information on the initiation of irreversible structural deterioration prior to rupture. Rather than replacing conventional seam strength measurements, the proposed parameter complements existing evaluation methods by characterizing an earlier stage of seam mechanical response that cannot be captured by the ultimate breaking force alone.

From an engineering perspective, this additional information may facilitate earlier assessment of seam mechanical integrity and support the optimization of sewing parameters during product development.

The introduction of the Remaining Load Capacity and Relative Yield Ratio provides additional insight into the mechanical behavior of stitched seams beyond the traditional breaking force analysis. While the breaking force describes only the ultimate failure point, the newly derived parameters quantify the mechanical reserve that remains available after the onset of localized seam yielding. Taken together, these findings indicate that the proposed Seam Yielding Point complements conventional seam strength measurements by providing an experimentally accessible means of identifying the initiation and progression of structural deterioration before ultimate seam failure.

## 5. Conclusions

This study systematically investigated the influence of sewing thread properties on seam mechanical behavior and introduced the Seam Yielding Point (SYP) as a complementary operational parameter derived from force–elongation curves. The experimental observations confirmed that seam performance is governed by the combined influence of sewing thread strength, fabric density, and weft yarn linear density. Increasing sewing thread strength and fabric compactness consistently resulted in higher seam yielding forces and higher ultimate seam strengths.

Analysis of the recorded force–elongation curves revealed the existence of a characteristic deformation stage preceding complete seam rupture. The proposed Seam Yielding Point (SYP), defined as the first localized decrease in tensile force following the initial elastic stage, was consistently identified in all investigated specimens. The repeatability of this behavior across different fabric structures indicates that the SYP represents a robust operational experimental indicator of the onset of irreversible structural deterioration within the stitched assembly.

A strong positive relationship was observed between seam yielding force and seam breaking force for all investigated fabric groups. Overall, the present investigation shows that the proposed SYP provides complementary information regarding seam mechanical behavior that cannot be obtained from conventional seam strength measurements alone. Rather than replacing existing evaluation methods, the proposed parameter extends the characterization of seam performance by identifying an earlier stage of structural degradation before ultimate failure.

From an engineering perspective, the proposed methodology may contribute to a more comprehensive assessment of seam mechanical behavior and support the optimization of sewing thread selection, seam design, and quality evaluation of sewn textile products. Future research should investigate the applicability of the proposed Seam Yielding Point under different fabric structures, fiber compositions, seam constructions, and industrial sewing conditions in order to further validate its usefulness as a complementary experimental parameter for seam characterization.

The proposed operational definition of the Seam Yielding Point establishes a foundation for future investigations aimed at improving the mechanical characterization of sewn textile assemblies beyond conventional ultimate strength measurements.

The additional quantitative analysis confirmed that the investigated seams retained a measurable load-bearing capacity after the occurrence of the Seam Yielding Point. Therefore, the proposed SYP parameter provides complementary engineering information that cannot be obtained from conventional breaking force measurements alone. Further work should evaluate the applicability of the proposed operational criterion to additional fabric structures, stitch types, seam configurations, and cyclic loading conditions. The findings presented in this study contribute to a more comprehensive understanding of seam mechanical behavior and provide an experimentally applicable framework for evaluating the initiation and progression of seam deterioration prior to ultimate failure.

## Figures and Tables

**Table 3:** Measurement results of fabric samples sewn with four different types of threads and a weft density of 25 cm^−1^.

Sample Marking	Thread Type	Seam Yielding		Breaking Point	
		Elongation (%)	Force (N)	Elongation (%)	Force (N)
A	K1	9.263	282	9.975	357
	K2	9.360	285	10.079	361
	K3	9.456	288	10.183	365
	K4	9.649	294	10.391	372
B	K1	8.108	226	8.814	306
	K2	8.279	231	9.000	312
	K3	8.364	233	9.092	316
	K4	8.535	238	9.278	322
C	K1	6.802	202	7.674	270
	K2	6.947	206	7.837	276
	K3	7.091	211	8.001	281
	K4	7.236	215	8.164	287

**Table 4:** Measurement results of fabric samples sewn with four different types of threads and a weft density of 17 cm^−1^.

Sample Marking	Thread Type	Seam Yielding		Breaking Point	
		Elongation (%)	Force (N)	Elongation (%)	Force (N)
D	K1	8.563	284	10.169	317
	K2	8.652	287	10.275	320
	K3	8.742	290	10.381	323
	K4	8.920	296	10.593	330
E	K1	7.780	217	8.486	263
	K2	7.943	221	8.665	269
	K3	8.025	223	8.754	271
	K4	8.189	228	8.933	277
F	K1	7.873	167	9.272	236
	K2	8.040	171	9.469	241
	K3	8.208	174	9.667	246
	K4	8.375	178	9.864	251

**Table 5:** Measurement results of fabric samples sewn with four different types of threads and a weft density of 10 cm^−1^.

Sample Marking	Thread Type	Seam Yielding		Breaking Point	
		Elongation (%)	Force (N)	Elongation (%)	Force (N)
G	K1	7.136	161	10.169	255
	K2	6.861	171	9.568	270
	K3	7.124	214	9.315	281
	K4	8.238	242	9.923	294
H	K1	5.994	116	8.814	201
	K2	7.221	160	9.388	213
	K3	7.081	183	8.897	227
	K4	7.630	191	9.119	244
I	K1	6.307	137	7.885	168
	K2	6.809	135	9.318	185
	K3	7.134	148	8.414	197
	K4	6.494	175	8.535	216

**Table 6:** Summary of the quantitative parameters derived from the Seam Yielding Point for different fabric structures.

Weft Density (cm^−1^)	Mean SYP (N)	Mean BP (N)	Mean RLC (N)	Mean RYR (%)
25 (Samples A–C)	242.6	318.8	76.2	75.9
17 (Samples D–F)	228.0	278.7	50.7	80.9
10 (Samples G–I)	169.4	229.3	59.8	73.9

## Data Availability

The original contributions presented in this study are included in the article. Further inquiries can be directed to the corresponding author.
